# A Compact Microwave Microfluidic Sensor Using a Re-Entrant Cavity

**DOI:** 10.3390/s18030910

**Published:** 2018-03-19

**Authors:** Hayder Hamzah, Ali Abduljabar, Jonathan Lees, Adrian Porch

**Affiliations:** 1 Engineering College, University of Al-Qadisiyah, Al-Qadisiyah, Diwaniyah 58001, Iraq; 2 Engineering College, University of Basrah, Basrah, Baghdad Street Qarmat Ali, IQ-61002, Iraq; aliaiq76@gmail.com; 3 Centre for High Frequency Engineering, Cardiff University, Wales, CF10 3AT Cardiff, UK; leesj2@cardiff.ac.uk (J.L.); porcha@cardiff.ac.uk (A.P.)

**Keywords:** re-entrant microwave cavity, microfluidic sensing, dielectric properties, segmented flow

## Abstract

A miniaturized 2.4 GHz re-entrant cavity has been designed, manufactured and tested as a sensor for microfluidic compositional analysis. It has been fully evaluated experimentally with water and common solvents, namely methanol, ethanol, and chloroform, with excellent agreement with the expected behaviour predicted by the Debye model. The sensor’s performance has also been assessed for analysis of segmented flow using water and oil. The samples’ interaction with the electric field in the gap region has been maximized by aligning the sample tube parallel to the electric field in this region, and the small width of the gap (typically 1 mm) result in a highly localised complex permittivity measurement. The re-entrant cavity has simple mechanical geometry, small size, high quality factor, and due to the high concentration of electric field in the gap region, a very small mode volume. These factors combine to result in a highly sensitive, compact sensor for both pure liquids and liquid mixtures in capillary or microfluidic environments.

## 1. Introduction

The re-entrant microwave cavity (RMC) is a very attractive sensor for dielectric characterisation of small (mL to µL) liquid volumes due to the high concentration of electric field in its gap region. The structure is also easy to manufacture, and retains a high quality (Q) factor (~3000) even when machined from aluminium. Unlike for the electric field, the associated magnetic field magnitude is small and spread over a much larger volume, leading to low surface loss on the exposed metal surfaces, hence the high Q. These desirable features (high Q, high concentration of electric field in the gap) contribute to a high performance dielectric sensor, as will be demonstrated.

There is a growing need for wireless sensing for critical industrial and military applications, medical/clinical applications, and structural health monitoring [1,2]. Microwave sensors, in particular, are of high interest in applications where no physical contact is possible or the use of active devices is impractical. They also offer numerous advantages compared to traditional techniques based on the conducting properties of a sample under test (SUT). The sensor presented here does not require any markers or labels and is therefore fast and non-invasive. Furthermore, such microwave sensors can be fully compatible with, and so can be embedded within, lab-on-a-chip type approaches [3].

Accurate microwave methods for dielectric characterisation (via measurement of the complex permittivity) are useful for applications in biomedicine and in pharmaceuticals. For example, bio-materials are often liquids and include blood, lymph, and other physiological solutions. Such bio-liquids, being mostly comprised of water, are characterized by high values of permittivity and moderate conductivity due to their electrolytic content. Hence, most bio-liquids have significant losses in microwave frequencies, arising mostly from the dipole relaxation of water at frequencies above about 2 GHz, or finite ionic conductivity due to dissolved electrolytes at frequencies below this [4].

**Figure 1:**
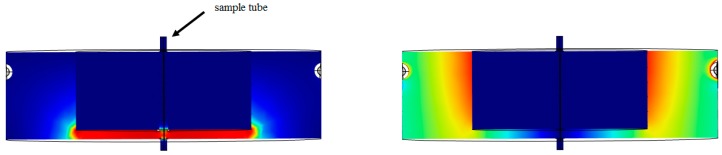
Cross section of a re-entrant microwave cavity, showing the distribution of electric (**a**) and magnetic (**b**) fields; the position of the sample tube within the axial hole.

Owing to the finite conductivity for bio-liquids it is important that the SUT does not make direct contact with any part of the sensor fixture. Many types of microwave cavity offer the potential of contactless measurements, such as single mode cylindrical/rectangular cavities and waveguides [5]. RMCs have been studied and used widely for a variety of applications, in radio and microwave communications systems [6,7], material dielectric properties measurements [8,9], and medical applications [10,11]. Their attractiveness is due to their simple mechanical construction and wide tuning range, with the narrow gap having the effect of reducing the frequency and focusing the electric field [12]. A cross section of the RMC is shown in Figure 1.

The interaction between an electric field and a dielectric sample by using microwave cavities has been studied widely, and the dielectric permittivity for a sample material can be measured by using perturbation theory where the sample is placed with its long dimension parallel to the electric field [13,14,15]. In [16], researchers measured the dielectric properties by using double split ring resonator when the sample in a perpendicular position with the electric field generated in the gaps, but greater sensitivity is obtained in the parallel orientation, which gives rise to the greatest electric dipole moment for a given applied electric field. A comparison study has been published [17] to achieve maximum electric field inside fluids when the channels which are carrying fluids have been placed in two orientations with an electric field, in both vertical and horizontal orientations.

In this work, we present a new approach by developing a miniaturized re-entrant cavity for studying the interaction between dielectric fluids and the electric field within the RMC’s gap region. Its simple geometry, wide frequency range tuning and high quality factor make it highly attractive for this application and it is also possible to study multiphase and segmented flow applications owing to the highly localized concentration of electric field energy. As can be seen from Figure 1, there is excellent separation between the regions of maximum electric and magnetic fields in a RMC [18,19]. For a dielectric sample placed on axis, there is then an unambiguous characterization of its dielectric properties.

## 2. Theory

Re-entrant cavities can be modeled using a lumped-element approximation in the limit when the gap dimension is small compared with the other dimensions of the cavity and with the resonant wavelength λo. The electric field concentrates in the gap between the centre post and the cavity wall and its direction from the inner post points to the cavity wall, perpendicular to the two surfaces of the gap thus formed. If the gap region is small, then the electric field in the gap will be approximately uniform and the magnetic field will be infinitesimally small near the axis. This region acts like a parallel-plate capacitor, its capacitance being inversely proportional to the gap width. The purely circumferential magnetic field is generated from surface currents flowing in the directions shown in Figure 1, with a displacement current across the gap. The largest magnetic field strength is near the short-circuit-end, at the opposite end of the gap (as shown in Figure 1) [18,19,20].

**Figure 2:**
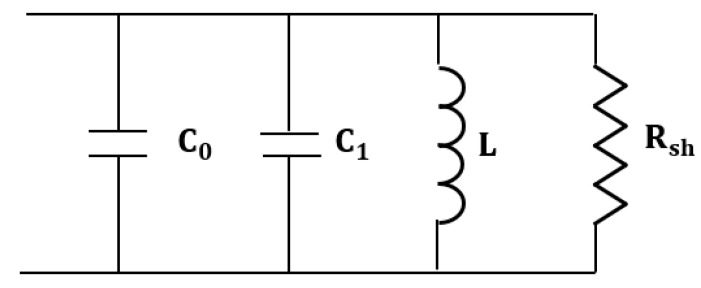
Equivalent lumped circuit for the re-entrant cavity.

The equivalent circuit of the lumped element mode of the RMC is shown in Figure 2, with C_0_ being the capacitance of the gap region and C_1_ the capacitance associated with charge leakage onto the outside surface of the central post [20]. The total equivalent capacitance of the cavity when operating in its lumped element mode is then [20]:

(1) $$C=C_{0}+C_{1}$$

**Figure 3:**
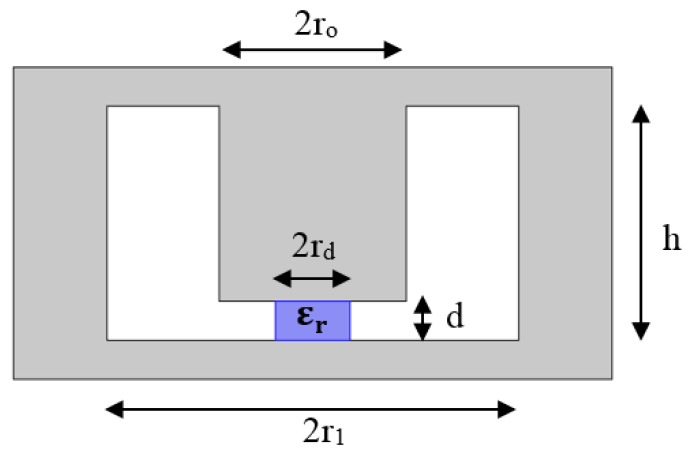
Re-entrant cavity with a gap partially filled with a dielectric.

Referring to the dimensions labelled in Figure 3, these two capacitances are approximately:

(2) $$C_{0}\approx \frac{{\pi \;r}_{0}^{2}\varepsilon_{0}}{d}$$

(3) $$C_{1}\approx 4r_{0}\varepsilon_{0}\ln\left(\frac{\sqrt{{\left(r_{1}-r_{0}\right)}^{2}+h^{2}}}{2d}\right)$$

The equivalent inductance L is:

(4) $$L\approx \frac{{\text{µ}}_{0}h}{2\pi }\ln\frac{r_{1}}{r_{0}}$$

where ${µ}_{0}$, and $\varepsilon_{0}$ are the permeability and the permittivity of free space, respectively. The equivalent shunt resistance is then [20]:

(5) $$R_{\mathrm{sh}}\approx \frac{2{\pi \delta \sigma \omega }^{2}L^{2}}{\left(\frac{h-d}{r_{0}}+\frac{h}{r_{1}}+2\ln\frac{r_{1}}{r_{0}}\right)}$$

where σ is the metal conductivity and δ is the skin depth. The resonant frequency $f_{r}$ and unloaded quality factor Q are, therefore, as usual for a lumped circuit:

(6) $$f_{r}=\frac{1}{2\pi \sqrt{\mathrm{LC}}},\;Q=\frac{R_{\mathrm{sh}}}{2{\pi f}_{r}L}$$

when a dielectric material inserted into the active region of the re-entrant cavity, which is the gap between the post and the cavity wall as shown in Figure 3, a new perturbed capacitance will be obtained [21]. For a narrow gap *d*, assumed to be much smaller than the central post radius *r_o_*, this increased capacitance can be calculated quite simply by application of the parallel plate capacitor formula for a composite capacitor consisting of capacitors in parallel, since the electric field is parallel to the air: dielectric boundary around the perimeter of the sample.

Assuming that the dielectric has a complex permittivity *ε* = *ε*_1_ − *jε*_2_, a simple approximate formulae for the cavity perturbation [14] by the sample can be derived, namely:

(7) $$\frac{\Delta f}{f_{r}}\approx -\frac{1}{2}\left(\varepsilon_{1}-1\right)\frac{V_{s}}{V_{eff}}$$

(8) $$\Delta \left(\frac{1}{Q}\right)=\frac{\Delta f_{B}}{f_{r}}\approx \varepsilon_{2}\frac{V_{s}}{V_{eff}}$$

where I, $\Delta f_{B}$ are the change in resonance frequency and bandwidth, respectively, owing to the presence of the sample and $f_{r}$ is the resonance frequency when the cavity is empty.

The quantity *Vs* is the sample volume and $V_{eff}$ is the mode volume of the cavity. This is the effective volume occupied by the electric field energy, which in the first approximation is the volume of the gap region but in practice is larger owing to the charge on the lower part of the central post (i.e., the “fringing field”).

## 3. Cavity Design and Fabrication

The key design strategy is twofold:

1. The electric field is parallel to the sample length, meaning that depolarization is minimal and the changes in resonator parameters (such as resonant frequency) are linearly dependent on the relative permittivity.
2. The effective volume of the re-entrant cavity is very small, yielding a sensitive sample characterization.

Furthermore, the COMSOL software has been used to optimise the sensor’s dimensions for the measurement of the samples described. COMSOL Multiphysics^®^ 4.4 (COMSOL Inc., Stockholm, Sweden) is a finite element analysis and multiphysics simulation platform that encompasses all of the steps in the modeling workflow, from model building, defining geometries, material properties, and the physics that describe specific phenomena to solving and producing accurate results. It was used in the cavity design to confirm a resonant frequency of approximately 2.4 GHz when the RMC is sample loaded, and in the determination of the fields needed to develop a more precise model for cavity perturbation. The wave equation in the frequency domain was computed in the electromagnetic wave model as described in the software as:

(9) $$\nabla \times \mu_{r}^{-1}\left(\nabla \times \bar{E}\right)-k_{0}^{2}\left(\varepsilon_{r}-\frac{j\sigma }{\omega \varepsilon_{0}}\right)\bar{E}=0$$

where $\mu_{r}$ is the relative permeability, $\varepsilon_{r}$ the relative permittivity and σ the electric conductivity of the material; $k_{0}$ is the wave number in free space, and *ω* the wave angular frequency. The impedance boundary condition is used for the aluminium surfaces in order to calculate their losses. The simulation boundary is the inside surface of the aluminium outer cavity. Two coaxial ports were used to feed the electromagnetic energy to the resonator, terminated in near-identical loop (i.e., inductive) coupling structures.

**Figure 4:**
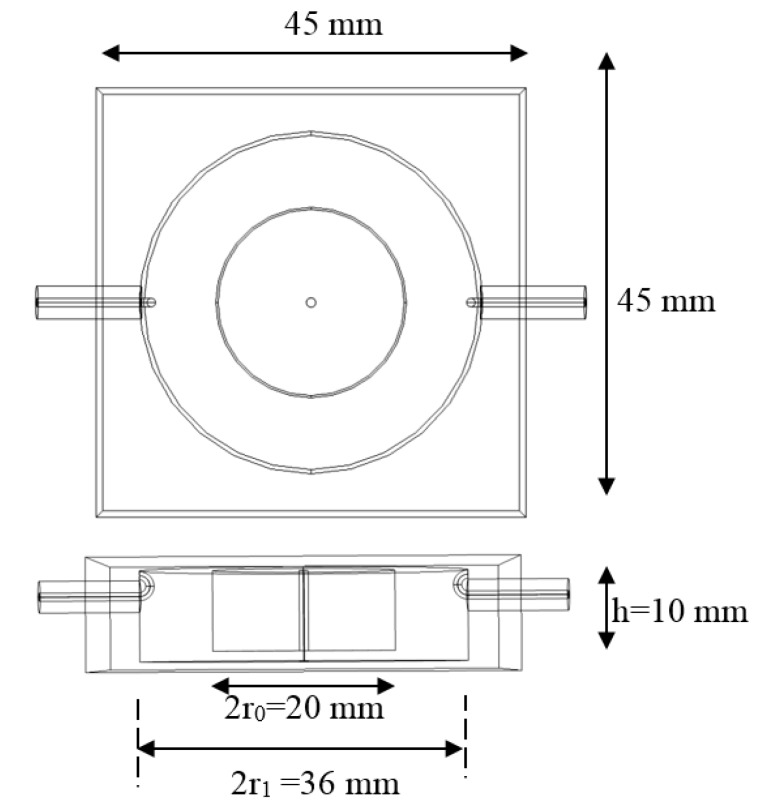
Re-entrant cavity dimensions.

In our constructed cavity, referring to Figure 3 and Figure 4, we choose *d* = 1 mm, *r*_1_ = 18 mm, *r_o_* = 5 mm and *h* = 10 mm. This gives a resonant frequency of the unperturbed RMC of around 2.43 GHz, within the ISM band for microwave heating and, hence, a common frequency for which dielectric characterization of materials is necessary. A 1 mm diameter hole has been drilled along the whole length of the central post to take a sample tube. Note the much smaller dimensions of the RMC compared with, for example, a cylindrical TM_010_ cavity, which would need to have an internal diameter of about 95 mm for the same resonant frequency. The RMC has a much smaller effective volume of two orders of magnitude smaller, about 0.8 cm^3^, compared with around 80 cm^3^ for the TM_010_ cylindrical cavity of the same frequency. 

**Figure 5:**
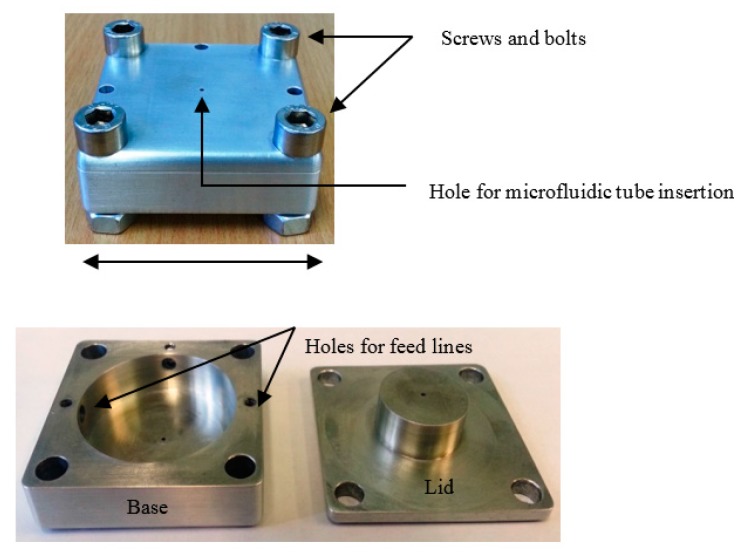
Photograph of the machined re-entrant cavity and its component parts.

Figure 5 shows the constructional diagram of the manufactured aluminium RMC, showing the base and lid. Two holes (3.6 mm diameter) have been made on the side surface for inserting the coaxial feed lines, made from RG402 coaxial cable with SMA connectors. The feedlines are terminated in small coupling loops that couple to the magnetic field. The magnetic field in the resonant mode is excited in the cavity when current flows on one of the loops, as per the simulation, whose excitation can be described as a magnetic dipole tangential to the cavity wall [22,23]. The coupling coefficients at both ports 1 and 2 are made equal by small adjustments to the loop angles and positions, so making the magnitudes of S_11_ and S_22_ approximately equal at resonance.

## 4. Microfluidic System Design

**Figure 6:**
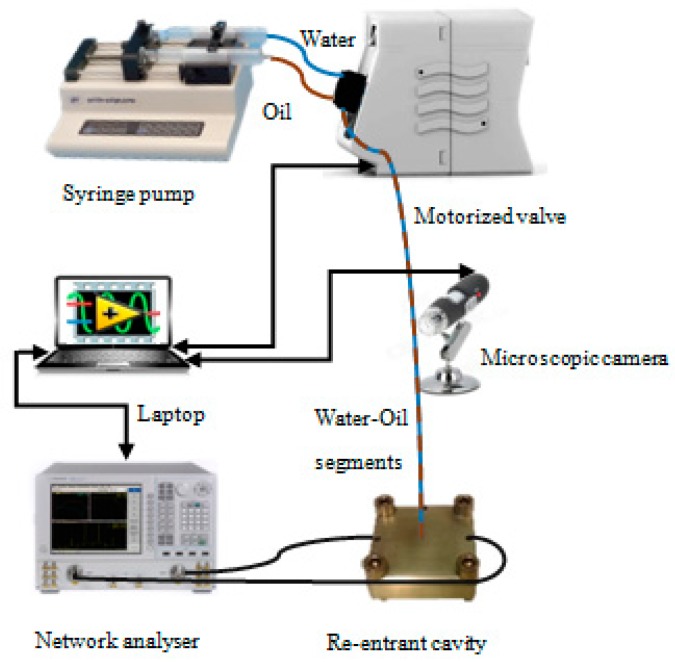
The complete microfluidic system connected to the cavity sensor and controlled via LabVIEW code. The microscopic camera has been used for video recording.

Fluorinated ethylene propylene (FEP) tubing of 0.77 mm O.D. × 0.4 mm I.D. has been used for the liquid sample flow through the RMC’s gap region. A programmable two-syringe pump (KD Scientific, Holliston, MA, USA)equipped with a motorized valve (MXX777-601) was used to control the flow of water and oil segments for the study of segmented flow, and also for pure samples of other common solvents. Microwave measurements of all S parameters in the frequency domain are recorded by using a vector network analyser (E507IB, Agilent, Santa Clara, CA, USA). All equipment is controlled by a laptop computer running LabVIEW code (National Instruments Ltd., Austin, TX, USA). A schematic of the whole measurement system is shown in Figure 6.

## 5. Results

### 5.1. Simulation Results

**Figure 7:**
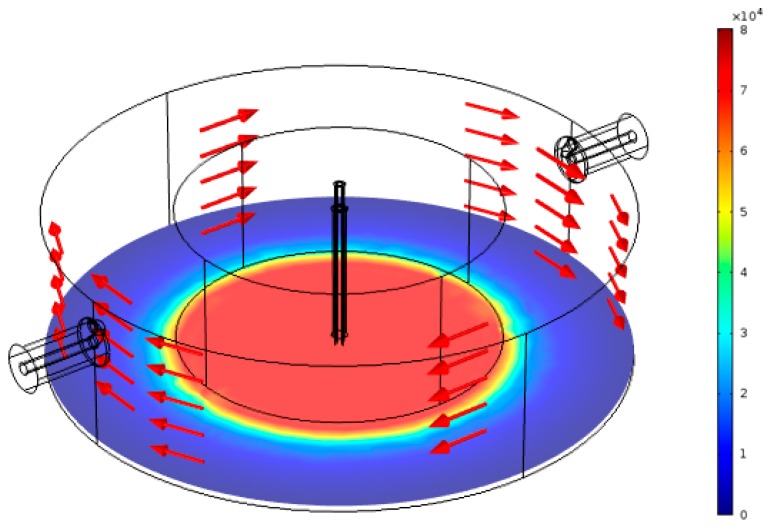
Simulation result for the normalized electric field (V/m) when the FEP sample tube is empty.

COMSOL Multiphysics has been used in the simulation and for calculating complex permittivity from the RMC measurements. Figure 7 shows the result when the FEP tube inside the sensor cavity is empty. It can be seen that, as expected, the electric field is concentrated almost uniformly in the gap area between the cavity post and end wall, while the magnetic field circulates in the space around the post.

**Figure 8:**
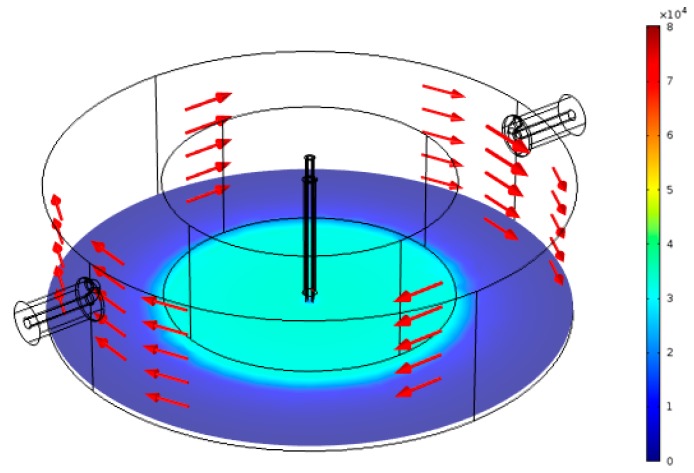
Simulation result for the normalized electric field (V/m) when there is water in the FEP tube within the active gap area.

The simulated fields for a water-filled FEP tube is shown in Figure 8. The absorbed power P per unit volume (W/m^3^) by a dielectric material is given by the frequency f of the applied electric field, the electric field strength E within the material, the material loss factor $\varepsilon_{2}$, and the free space permittivity $\varepsilon_{0}$ [24,25]:

(10) $$P=2\pi fE^{2}\varepsilon_{0}\varepsilon_{2}$$

**Figure 9:**
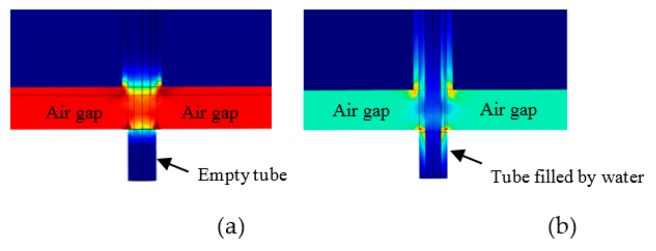
Cross sectional view when FEP tube is empty (**a**); and when there is water in tube in the active gap area (**b**).

**Figure 10:**
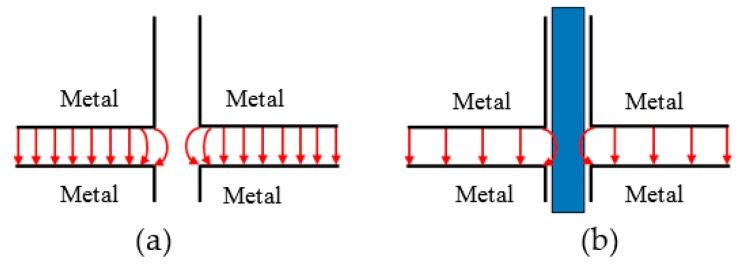
Schematic diagram of the electric field lines near the sample when (**a**) the cavity is empty; and (**b**) when there is a water-filled tube.

An expanded view of the cavity’s cross sectional area is shown in Figure 9, when the cavity is empty (a), and when there is water in the tube (b). A schematic of the resulting electric field lines is shown in Figure 10. The component of electric field along the tube is continuous across the boundaries, and this boundary condition gives rise to a large electric field inside the water sample for large cavity perturbations, leading to a sensitive measurement of sample dielectric properties. 

### 5.2. Experimental Results

**Figure 11:**
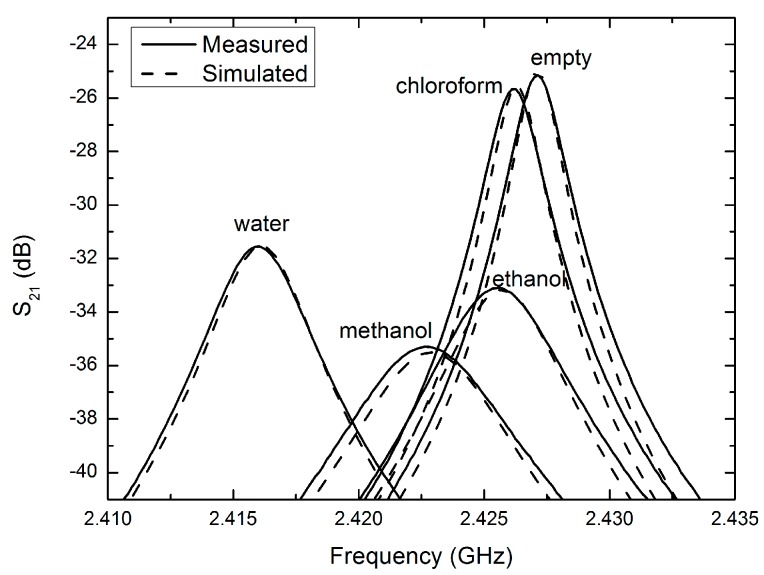
Measured and simulated |S_21_| for several solvents at 25 °C.

Figure 11 shows very good agreement between the simulated and experimental results when the resonator is empty, and then when the tube is filled with common liquids. The Debye model Equation (11) has been coded into COMSOL to determine the complex permittivity of the liquids to generate these simulation results:

(11) $$\varepsilon \left(\omega \right)=\varepsilon_{\infty }+\frac{\varepsilon_{s}-\varepsilon_{\infty }}{1+j\omega \tau }$$

Here, $\varepsilon_{s}$ is the permittivity in the static limit (ωτ << 1), $\varepsilon_{\infty }$ is the permittivity in at very high frequencies (ωτ >> 1) and τ is the relaxation time. The Debye parameters $\varepsilon_{s}$, $\varepsilon_{\infty }$ and τ are taken from [16] and are presented in Table 1 at room temperature.

**Figure 12:**
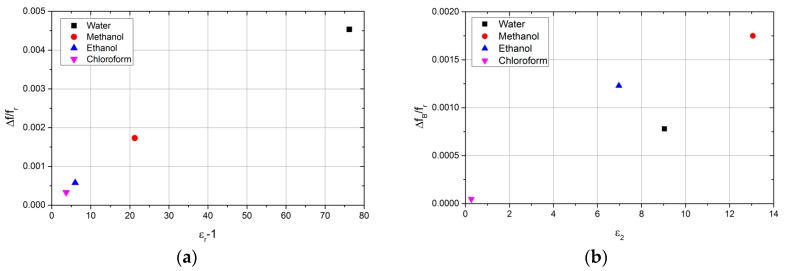
(Δ*f*/*fr*) for water, methanol, ethanol, and chloroform vs *ε*_1_-1 (**a**); and (Δ*f_B_*/*f_r_*) for the same solvents vs. *ε*_2_ (**b**).

Table 2 illustrates the simulated and experimental values for resonant frequency, quality factor, and insertion loss, for a RMC with a FEP sample tube filled with a selection of common solvents. From these measured values, the relative permittivity has been extracted using COMSOL and compared with simulated values. The small error ratios normalized by the unperturbed resonant frequency, have been plotted as functions of real and imaginary parts of the complex permittivity; the results are shown in Figure 12a,b, respectively, where f_r_ is the resonant frequency and f_B_ is the bandwidth. The fact that the frequency shift data of Figure 12a gives an approximately linear dependence on the real part of the permittivity is an important result that indicates that there is little depolarisation in this sample geometry, with the electric field parallel to the sample tube. This ensures full sensitivity of the resonator to changes in the permittivity of the sample liquid even for high values of permittivity. Conversely, if the electric field is perpendicular to the sample tube, the frequency shift saturates for high values of real permittivity, greatly compromising the sensitivity of the resonator in this regime.

### 5.3. Results for Segments Flow

**Figure 13:**
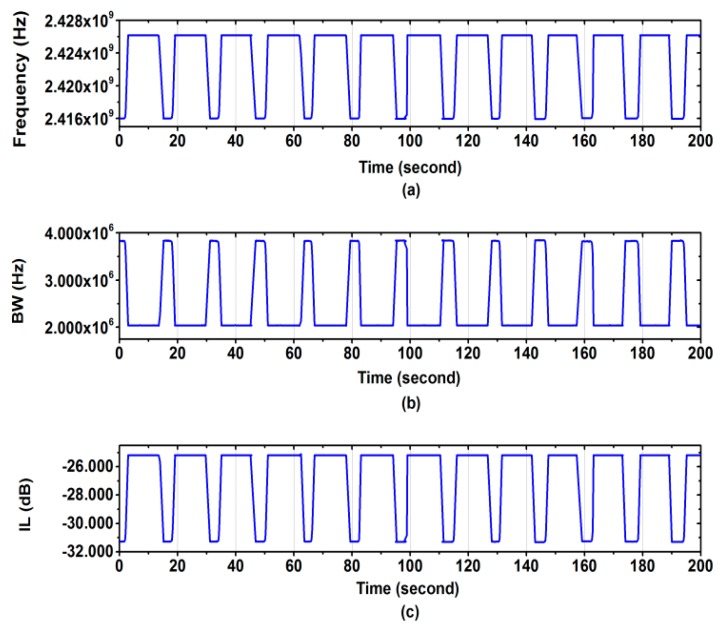
RMC perturbations due to a segmented oil: water flow. (**a**) resonant frequency (water = low frequency); (**b**) bandwidth (water = high bandwidth); and (**c**) insertion loss (water = high loss).

The compact RMC has been tested for a flow involving different lengths of segments of water and oil. The system used is described in the Section 4. Figure 13 shows how the resonant frequency, bandwidth (BW), and insertion loss at resonance (IL) vary with the segment type present within the gap region (water segment length is 18.5 mm, and oil segment length is 35.5 mm). There is excellent contrast between the segment materials, in all three of these resonator parameters. Note that there are only two independent measurement variables here, since BW and IL both quantify the same thing (i.e., microwave loss, or *ε*_2_). This contrast is enabled due to the RMC’s sensitivity to the sample within the gap region, giving a highly localised measurement of sample permittivity.

By using a microscopic camera, the segment lengths and times required to travel through the gap region have been recorded. The length of segment can be calculated from the velocity and the required time to pass through the active gap as shown in Table 3. Segmented flows are important, for instance, in analytical and quality control procedures.

## 6. Conclusions

In this paper, a compact re-entrant microwave cavity has been designed and manufactured. COMSOL Multiphysics software has been used in the design and in measurements, to compare theoretical and experimental results. The re-entrant cavity is designed to work as a sensor and there is excellent agreement between simulated and experimental results when the cavity is tested with some common liquids. Also, the sensor’s ability to characterize the dielectric properties and flow parameters for segmented flow has been tested for short water-oil segmentation.

Future work includes assessing the ability of this cavity to coupling strong electric fields into a small, well-defined sample region (within the gap region) for disruption and heating purposes. This supports parallel studies investigating the use of microwave methods for the rapid extraction of DNA from certain types of antimicrobial resistant bacteria (e.g., *C. difficile*).

## Figures and Tables

**Table 1:** Debye model parameters for liquids at room temperature.

Resonator	$\varepsilon_{s}$	$\varepsilon_{\infty }$	τ (ps)
Water	78.4	5.16	8.27
Methanol	32.5	5.60	51.5
Ethanol	24.3	4.20	163
Chloroform	4.72	2.50	7.96

**Table 2:** Summary of results.

Resonator	Simulated f_r_ (GHz)	Measured f_r_ (GHz)	Simulated Quality	Measured Quality	Simulated IL (dB)	Measured IL (dB)	Simulated Permittivity	Measured Permittivity	Error
Empty	2.4271	2.4271	1190	1187	−25.020	−25.153			
Water	2.4161	2.4160	615	613	−31.527	−31.578	77.23 − j9.04	77.85 − j9.10	0.8%
Methanol	2.4229	2.4227	385	381	−35.509	−35.296	22.27 − j13.06	22.84 − j12.87	1.5%
Ethanol	2.4257	2.4255	483	480	−33.169	−33.117	7.01 − j6.97	7.27 − j6.96	1.7%
Chloroform	2.4263	2.4261	1125	1125	−25.557	−25.663	4.69 −d j0.27	4.81 − j0.30	2.5%

**Table 3:** Water and oil segment length measured by camera and RMC.

Segment Type	Velocity (mm/sec.) Measured by Camera	Length (mm) from Camera	Length (mm) from Cavity
Water	3.4 ± 0.2	18.5 ± 1.2	18.2 ± 0.9
Oil	3.4 ± 0.2	35.5 ± 1.2	33.6 ± 1.6
